# Structural Basis for S100B Interaction with its Target Proteins

**DOI:** 10.4172/1747-0862.1000366

**Published:** 2018-09-10

**Authors:** KD Prez, L Fan

**Affiliations:** Department of Biochemistry, University of California Riverside, 900 University Ave, Riverside, California, USA

**Keywords:** Neurological diseases, Cancers, S100B-binding partners, p^53^ Tumor suppressor, Transcription factor TFIIH, XPB helicase, DNA repair

## Abstract

The S100B protein is an intra- and extracellular signaling protein that plays a role in a multitude of cellular processes and abnormal S100B is associated with various neurological diseases and cancers. S100B recognizes and binds effector proteins in a calcium-dependent manner. S100B has been shown to interact with the actin capping protein CapZ, protein kinase C, Hdm2 and 4, RAGE receptor, and p^53^, among others. These protein partners interact with a common area on the S100B protein surface, validating the method of using the consensus sequence for S100B target search. In addition, each S100B target protein distinguishes itself by additional contacts with S100B. This perspective suggests that the combination of sequence homology search and structural analysis promises to identify newer S100B-binding partners beyond the use of the consensus sequence alone as the given example in the XPB subunit of the TFIIH general transcription factor. XPB is a helicase required for both transcription and DNA repair. Inherited xpb mutations are associated with human disease Xeroderma Pigmentasum, Cockayne syndrome, and trichothiodystrophy. S100B protein is likely associated with much more biological pathways and processes. We believe that S100B will attract more and more attentions in the scientific community and S100B related studies will have important implications in human health and medicine.

## Introduction

The S100B protein belongs to the S100 family of Ca^2+^-binding signaling proteins which share dual conserved calcium-binding EF- hand motifs. S100 proteins exist exclusively in vertebrates, with 24 members observed in humans [1]. S100B is expressed in astrocytes, Schwann cells, melanocytes, chondrocytes, and adipocytes, among others [2].

Acting as both an intracellular regulator and as a secreted signaling molecule, the S100B protein plays a role in a myriad of cellular processes, including cell proliferation, migration, apoptosis, and differentiation [3–7].

Consequently, aberrant expression levels of S100B have been implicated in a variety of neurological diseases, cancer, and inflammatory disorders [8–14]. As S100B has no intrinsic enzymatic activity, its intra and extracellular functions are achieved exclusively by physical interactions to its target molecules in a calcium-dependent manner.

Currently S100B has been reported to interact with a variety of protein targets including the p53 tumor suppressor, CapZ, the RAGE receptor, NDR kinase, neurotensin, cathepsin L inhibitor, Hdm2, Hdm4, protein kinase Cα, ROS-GC1, microtubule-associated tau proteins, melittin, amyloid-β, interleukin-11, the serotonin 5-HT7 receptor, the dopamine D2 receptor and RSK1 [15–29]. This short perspective focuses on the structural basis of S100B-protein interactions.

## Literature Review

### Structural comparison of S100B-peptide complexes

**Figure 1:**
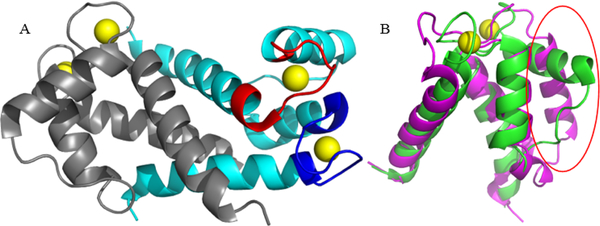
S100B dimer and Calcium induced conformational changes. (A) S100B Ca^2+^-bound dimer in ribbon representation (PDB entry 2h61). Monomers are colored in grey and cyan. The C- terminal EF-hand motif of the cyan monomer is colored red, while the N-terminal pseudo-EF-hand motif is colored blue. EF-hand bound Ca^2+−^ ions are shown as yellow spheres. (B) Alignment of apo-S100B (magenta; PDB entry 1b4c) and Ca^2+−^ bound (green; PDB entry 2h61) S100B, shown as in A. Ca^2+−^ induced Helix 3 rearrangement is highlighted with a red oval.

S100B exists primarily as a homodimer of two approximately 11 kDa monomers (Figure 1A), though stable and active tetrameric, hexameric, and octameric forms have been reported [30]. The S100B monomer consists of four α-helices with a β-strand between both helices 1 and 2, and 3 and 4, composing two helix-loop-helix EF-hand motifs connected by a linker region. The C-terminal canonical motif is made up of 12 amino acids involved in Ca^2+^-ion binding, while the N- terminal EF-hand (also termed the “S100-hand” ) contains 14 amino acids, therefore being considered by some as a pseudo-EF-hand because of the extra two amino acids [31,32]. Calcium binding in the N-terminal site I induces limited changes to the structure as a whole (RMSD = 1.472 over 33 atoms), while binding at the C-terminal site II induces a conformational change in helix 3 of up to 90°, leading to the exposure of the binding site for target proteins to access (Figure 1B) [33,34].

S100B interacting protein targets are largely identified by a sequence-based approach using the consensus S100B-interacting sequence ([K/R]-[L/I]-x-W-x-x-I-L). Pioneered by Ivanenkov et al. [18], the sequence was initially proposed based on phage display library screening for bacteriophage exhibiting Ca^2+−^ dependent interaction with S100B. Employing the consensus sequence in homology searches revealed the actin capping protein (CapZ) as a potential target [18]. Specifically, the peptide TRTKIDWNKILS, referred to as TRTK-12, was chosen due to its significant homology to the consensus sequence. Further investigation revealed Ca^2+−^ dependent interaction between S100B and TRTK-12 or CapZ, leading to the TRTK-12 inhibition of the S100B-CapZ interaction [18]. Subsequent expansion of the S100B-interaction consensus sequence by Wilder et al. to [K/R]-[L/I]-[P/S/N/D]-[W/L/I]-[S/D/L]- x -[L/I]-[L/F] allowed for additional protein targets to be identified and analyzed for S100B interaction. S100B has so far been shown to interact with short peptides derived from protein sequences of the p^53^ tumor suppressor, CapZ, the RAGE receptor, NDR kinase, neurotensin, cathepsin L inhibitor, Hdm2, Hdm4, protein kinase Cα, ROS-GC1 [21,22], microtubule-associated tau proteins, melittin, amyloid-β, interleukin-11, the serotonin 5-HT7 receptor, the dopamine D2 receptor and RSK1 [15,19–29].

The NMR structure solution of the bovine S100B apo-form yielded the first structural view of a S100B protein [35]. Since then, the past 20 years has seen a wealth of information on S100B conformational variability induced by pH [36], temperature [37], and metal ion- dependence [30,33,38–41]. S100B primarily interacts with its target proteins in the Ca^2+−^bound state [42]. Other targets, such as the giant phosphoprotein AHNAK, are recognized in a Zn^2+−^ dependent manner [43]. Several complexes of Ca^2+−^ bound S100B with short peptides derived from its known targets have been evaluated by NMR and X-ray crystallography, including p^53^, the NDR kinase, the RAGE receptor, RSK1 and CapZ [19,29,43–48].

The S100B interaction with p^53^ was first proposed by Baudier et al. based on the similarity of the p^53^ PKC-phosphorylation site to that of the myristoylated alanine-rich C kinase substrate (MARCKS) protein [49]. MARCKS phosphorylation is inhibited by EF-hand proteins, including S100B, though the MARCKS PKC-site is not in a good agreement with the S100B consensus sequence [50]. In addition, another region of p^53^ capable of interacting with S100B was identified in the p^53^ oligomerization domain [17]. S100B thus inhibits p^53^ oligomerization [4], as well as phosphorylation by PKC at the p^53^ C- terminus [49]. It was further shown that a peptide derived from the p^53^ carboxy-terminal regulatory domain (CTD) could be phosphorylated by PKC, and this activity could be inhibited by S100B [16]. Such inhibition events consequently reduce the p^53^ transcriptional activity, preventing its stimulation of cell cycle arrest and apoptosis [4].

To counteract the deleterious effects of S100B on p^53^ activity, small molecule screening studies have revealed several inhibitors of the S100B-p^53^ interaction including pentamidine (Pnt) in the form of pentamidine isethionate, an antiprotozoal drug currently approved for treatment of Pneumocystis cariini pneumonia [51,52]. Pnt has been shown to disrupt the S100B-p53 complex, resulting in increased cell apoptosis, p^53^ expression, and decreased cell migration [53]. Phase II clinical trial results revealed a myriad of adverse effects during melanoma treatment with Pnt (www.clinicaltrials.gov, identifier NCT00729807). Structure solution of the S100B-Pnt complex showed two Pnt molecules bound per monomer, occupying two sites adjacent to the p^53^ binding surface [40]. Further screening studies found several potential inhibitors binding in the hydrophobic cleft of the p^53^ binding site [52,54]. These findings together show three binding sites for drug inhibitors of the S100B-p53 interaction. Currently, the goal is to design inhibitors that span all three sites, likely providing higher affinity and specificity for S100B binding. The Weber group has performed several studies investigating small molecules binding in the three different sites and identifying the so called “FF-gate” composed of Phe87 and Phe88, normally occupying a channel between sites 1 and 2, occluded by a Pnt analog [52,55–57]. They also identified molecules exclusive to site 3 [56]. With the extensive structural and biochemical data available, a potent inhibitor of the S100B-p53 complex formation seems right around the corner.

## Discussion

**Figure 2:**
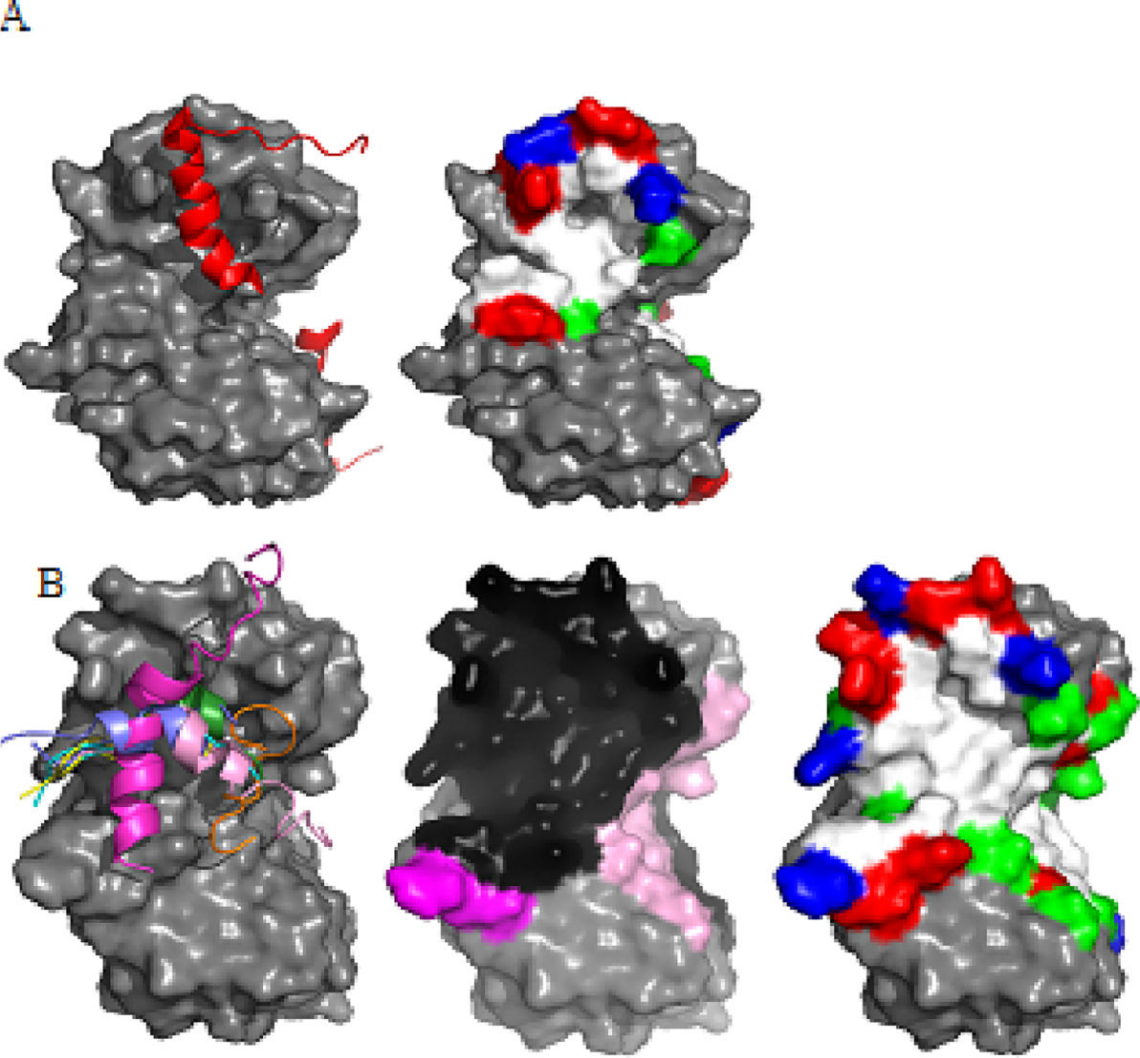
Structural comparison of the S100B-peptide interfaces. (A) The S100B-p^53^ peptide complex (PDB entry 1dt7). (Left) S100B is shown as a gray surface with the p^53^ peptide in cartoon representation (red). (Right) Residue properties of the p^53^-binding pocket (white – hydrophobic, green – polar, blue – basic, red – acidic). (B) Common S100B-peptide interaction surface. (Left) S100B from the NDR-kinase model (PDB entry 1psb) shown as a gray surface with the peptides of NDR (magenta), RAGE (green; PDB entry 4xyn), RSK1 (pink; PDB entry 5csn, blue; PDB entry 5csj, yellow; PDB entry 5csi, cyan; PDB entry 5csf), and TRTK-12 (orange; PDB entry 1mq1) based on superposition of the S100B C- terminus (aa 29–88). (Middle) S100B dimer surface colored accordingly to the bound peptides on the left with common-binding area in black. (Right) Residue properties of the peptide-binding pocket (white – hydrophobic, green – polar, blue – basic, red – acidic).

Structural analysis of the complex between S100B and a p^53^-CTD peptide (amino acids 367–388) revealed an induced folding of the peptide, normally unstructured, into an α-helical structure (Figure 2A) [44]. This same induced helical fold is observed in other targets of S100B: RSK1, RAGE, and the NDR kinase (Figure 2B). While all three of these peptides adopt a similar fold when bound to S100B and share a common binding area on the surface of S100B, each target protein does distinguish itself by additional contacts with S100B. In the complex of S100B with an NDR kinase peptide, several hydrophobic contacts are made between NDR side chains and the hydrophobic core of the S100B binding site [19]. In addition, electrostatic interactions are observed between helical side chains from NDR with the linker region of S100B [19]. In the S100B-RSK1 study, Gogl et al. used several peptides to generate co-crystals of the protein-peptide complex, resulting in several crystal structures showing altered binding of peptide(s) to S100B dimers [29]. These interactions have been confirmed by SAXS analysis and NMR NOE assignments [29]. Two of the four tested peptides adopted a helical fold extending through the binding pocket of S100B, with one nearly extending into the unbound S100B subunit of the dimer (Figure 2B). Both structures, however, seem to bypass completely the canonical hydrophobic binding pocket of Ca^2+^-bound S100B, with primarily hydrogen bonding and electrostatic interactions with the S100B linker and surface residues facilitating the interaction. Binding of the RAGE peptide, on the other hand, relies almost exclusively on the S100B hydrophobic binding pocket [45]. With minimal hydrogen bonding and no clear salt bridge formation between the peptide and S100B, the interaction is maintained via burying three hydrophobic residues in the S100B site after the induced helical fold of the peptide (Figure 2B). Indeed, the diversity observed in the interaction surfaces among S100B and its target proteins reveals an extremely large binding surface on S100B allowing for binding a diverse selection of peptide sequences (Figure 2B) [15].

To quantitatively compare the interaction energies of the S100B- peptide complexes, we analyzed the structure models using the Proteins, Interfaces, Structures and Assemblies (PISA) server [58]; the results are summarized in Table 1. The results of the PISA analysis compared to the dissociation constants reported for the different complexes reveal a strong correlation between the ΔG of solvation and the Kd values. One exception was the TRTK-12 peptide, though this is likely due to the exceptionally hydrophobic nature of its interface as evidenced by its low ΔG P-value. Indeed, the strongest interaction (Kd = 0.04 ± 0.02 μΜ) coincides with the largest ΔG (−13.4 kcal/mol) in the RSK1-A peptide while the weakest interaction (K_d_ = 23.5 ± 6.6) has the smallest ΔG (−5.0 kcal/mol) in the p^53^ peptide-S100B complex.

This apparent correlation between the *in silico* and *in vitro* quantitative data suggests that known structures can be used as the guidance to identify new S100B targets of high-affinity. As the pool of available S100B-target peptide complex structures grows, it has become clear that the S100B-interaction consensus sequence is largely limiting in that the specific residues involved in interaction with S100B vary greatly between peptide sequences [15]. To reflect this in the search for new S100B targets, specific peptide sequences should be used together with structural homology of the interacting residues. Utilizing this new approach, we identified a new potential target of S100B in the XPB helicase subunit of the general transcription factor TFIIH.

### XPB-S100B proposed interaction

The Xeroderma pigmentosum complementation group B (XPB) helicase is the largest subunit of the general transcription factor II (TFIIH) complex. XPB plays vital roles in both transcription and nucleotide excision repair [59]. Being an ATP-dependent 3̛-5̛ helicase, the XPB helicase facilitates the opening of the DNA helix during NER to allow for removal of bulky DNA adducts generated as a consequence of UV exposure or chemical therapies for anticancer treatment [59,60]. In addition, the XPB ATPase activity is critical for initiation and promoter melting during transcription [61–63].

Several structural studies have provided a framework for how XPB functions as a molecular wrench to melt dsDNA both alone [64,65] and in the context of TFIIH [66–68]. In addition, a high-resolution crystal structure by Hilario et al. provided a model for how the proposed XPB-XPF complex forms to facilitate the 5̛ -incision during NER [69]. Phosphorylation of Ser751 in the C-terminus of XPB acts as a key regulatory site during NER via modulating XPF activity [70]. Furthermore, we notice that the XPB extreme C-terminus shares sequence homology with key residues of the p^53^-CTD, and so we propose that XPB should interact with S100B in a similar fashion to that of the p^53^-S100B complex [44]. Interestingly, such interaction was postulated previously by Lin et al. based on the XPB-p^53^ C-terminal interaction [4]. XPB could block the inhibitory S100B targeting of p^53^ in a competitive manner. A similar mode of regulation was proposed for the S100B-RSK1 complex by Gogl et al. Based on their structural modeling, S100B binding could block ERK2 binding to RSK1, and thus inhibit the phosphorylation of the RSK1 activation loop [29]. This inhibition of RSK1 phosphorylation by S100B, coupled with its inhibition of the RSK1 C-terminal kinase domain, explains how S100B blocks the MAPK signal cascade via RSK1 in malignant melanoma [29]. In a similar fasion, S100B-XPB interactions could have a regulatory role in transcription and/or DNA repair. Specifically, S100B binding to the XPB C-terminal tail could interfere with the phosphorylation of Ser751 of XPB and/or dephosphorylation, resulting in inhibition or stimulation, respectively, of the XPF endonuclease during DNA repair [70]. Alternatively, S100B could physically block the interaction of XPB with the XPF endonuclease complex directly.

**Figure 3:**
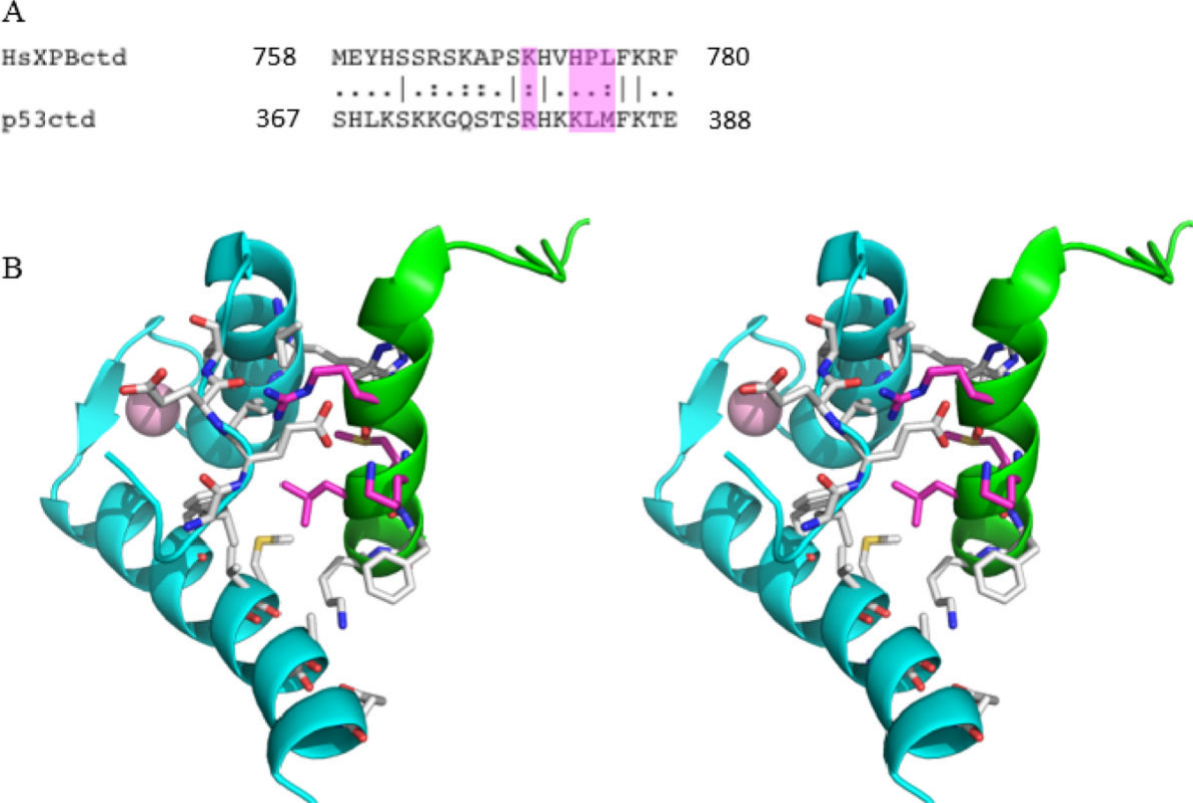
The XPB-S100B interaction likely resembles the p53- S100B complex. (A) A BLAST sequence alignment of the XPB C- terminus with the p53 negative regulatory domain. Unconservative interacting residues are highlighted in magenta. (B) Stereo-view of the S100B (cyan) complex with p53 (green) interacting region (PDB entry 1DT7) shown in cartoon representation, with residues composing the interface displayed in sticks. Unconservative interacting residues of p53 with XPB are shown with carbons in magenta. The bound calcium ion is shown as a pink sphere.

**Figure 4:**
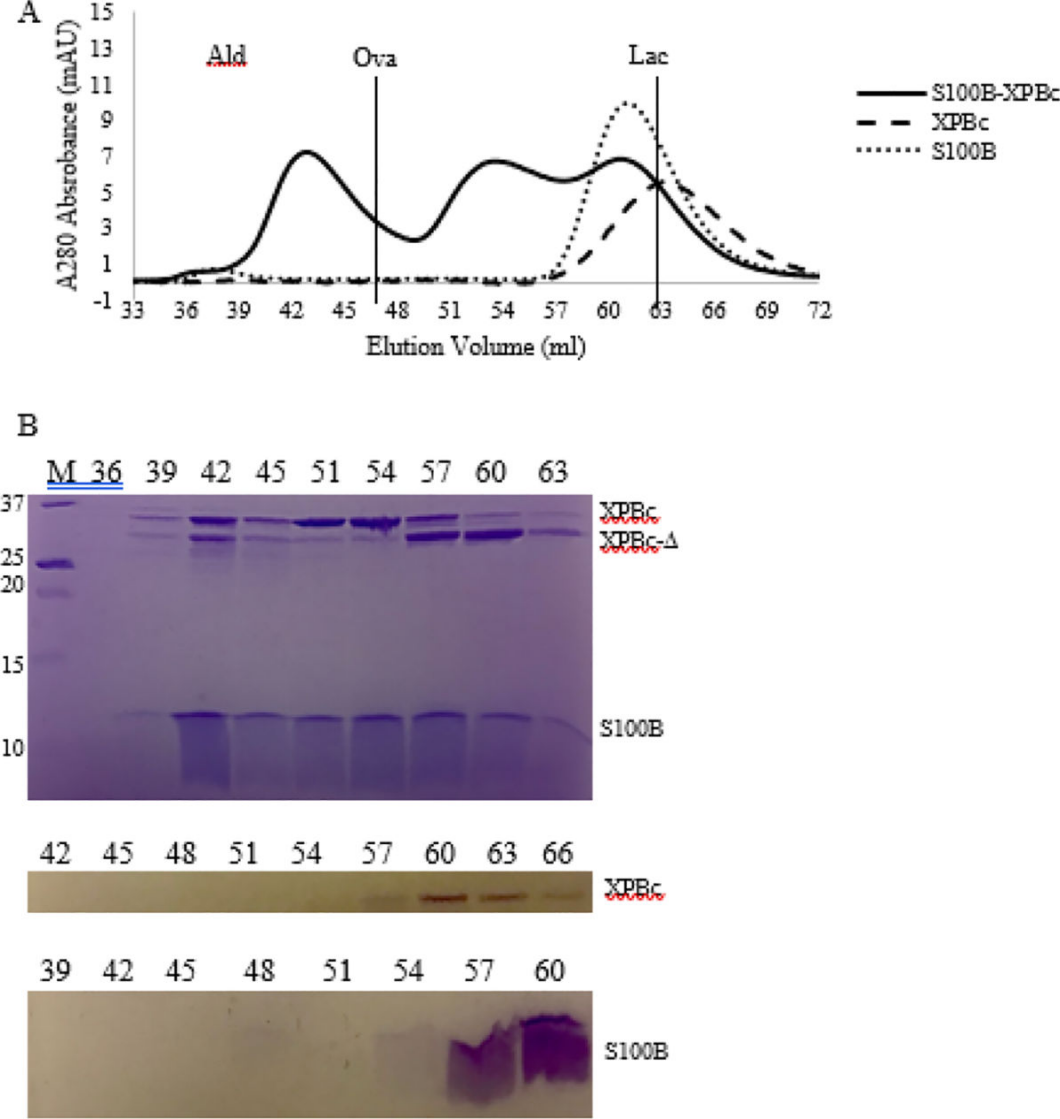
Interaction of XPB with S100B. (A) Gel filtration chromatography profile of the XPB C-terminal half (amino acids 494–782) alone (dashes), S100B dimer alone (dots), or the mixture of the two (solid). The peak shift of the mixed sample compared to those of XPBc and S100B indicates the formation of a stable S100B- XPBc complex. The calibration protein elution positions, demarcated by vertical lines for clarity, were generated from a mix of aldolase (158 kDa, Ald), ovalbumin (44 kDa, Ova), and lactalbumin (14 kDa, Lac) protein standards. (B) SDS-PAGE analysis of the chromatography profiles in (A). Protein bands – XPBc (494–782); XPBc-Δ (494–730, XPBc degraded during purification [69]); M – marker, # – elution volume fraction.

Although the amino acid sequences of the XPB and p^53^ extreme C-termini share low sequence homology (~23% using BLAST), most of the conserved residues in p^53^ form direct contacts with S100B in the S100B-p^53^ peptide complex (PDB entry 1DT7) (Figures 3A and 3B). Specifically, Arg379, His380, Lys382, Met384, Phe385, and Lys386 of the p^53^ peptide show direct contacts with residues of S100B, implying similar interactions in the S100B-XPB complex involving the identical or similar residues Lys771, His772, His774, Leu776, Phe777, and Lys778 of XPB, respectively. Our preliminary results do suggest a stable complex formed between S100B and the XPB C-terminal half consisting of residues 494–782 (Figure 4). During gel filtration chromatography, the peaks of individual XPB and S100B are both shifted to a higher apparent molecular weight when the two proteins are mixed together (Figure 4A). Furthermore, we noticed that a degraded XPB protein (XPBc-Δ) lack of the last 52 residues did not shift, in agreement with our prediction that XPB interacts with S100B via its C-terminal residues. These observations confirm that the sequence conservation between the C-termini of XPB and p^53^ seems sufficient for the interactions with S100B. However, one important discrepancy lies with Leu383 of p^53^, as the corresponding residue of XPB is Pro775. The presence of a proline residue in the XPB sequence would prevent formation of an alpha-helical structure in this region of XPB due to the geometric restrictions of the proline side-chain. This would suggest some changes in the S100B-XPB interface compared to that of S100B-p53 complex. It will be very interesting to further investigate the structural features of the S100B-XPB interface and the biological impact of such an interaction (Figure 4B).

## Conclusion and Future Research

In this review, we analyzed the structural basis of the S100B-peptide interactions on available data and literature information. Besides the common binding area shared by all S100B interacting proteins, the additional contacts provided by each target warrant the limitation of sequence only search in identifying new S100B target proteins. We believe that the combinatorial sequence/structure-homology approach for S100B target identification will expand new S100B targets beyond the use of sequence-based searches alone. The identification of the XPB protein as a new S100B target likely reveals a new means of regulating transcription and/or DNA repair through S100B. With the diversity of targets already identified, the processes involving S100B, and other S100 proteins, are likely far more extensive than what we currently know and could have important implications in human health and medicine.

## Figures and Tables

**Table 1:** PISA analysis of S100B-peptide complex structures.

PDB Entry	Peptide		Interface Area (A2)	ΔG (kcal/mol)	ΔG P-value	K_d_ (ΔM)	Reference
1dt7	p53 (367–388)	SHLKSKKGQSTSRHKKLMFKTE	480.0^b^	−7.3^b^	0.328^b^	23.5 +/− 6.6	[44]
1psb	NDR (62–87)	KRLRRSAHARKETEFLRLKRTRLGLE	689.2^b^	−9.3^b^	0.328^b^	20 +/− 10	[19]
4xyn	RAGE (54–68)	NTGRTEAWKVLSPQG	466.9	−8.7	0.253	2.7 +/− 0.5	[45]
1mq1	TRTK-12 (265–276)	TRTKIDWNKILS	525.7^b^	−9.9^b^	0.131^b^	0.27 +/− 0.03	[48]
5csf	RSK1-A (683–735)	QSQLSHQDLQLVKGAMAATYSALNSSKPTP QLKPIESSILAQRRVRKLPSTTL	590.4^c^	−13.4^c^	0.465^d^	0.04 +/− 0.02	[29]
5csi	RSK1-A’ (689–735)	QDLQLVKGAMAATYSALNSSKPTPQLKPIES SILAQRRVRKLPSTTL	716.0^c^	−12.1^c^	0.491^d^	1.8 +/− 0.3	[29]
5csj	RSK1-B (696–735)	GAMAATYSALNSSKPTPQLKPIESSILAQRR VRKLPSTTL	583.9^c^	−12.2 c	0.464^d^	2.5 +/− 0.2	[29]
5csn	RSK1-C (683–720)	QSQLSHQDLQLVKGAMAATYSALNSSKPTP QLKPIESS	714.0^c^	−9.2 c	0.632^d^	9.6 +/− 1.4	[29]

a The amino acids are color coded as follows based on the PISA analysis: Red – interfacing residue with S100B; black – non-interfacing residue; grey – not modeled in the PDB Entry.

b Mean value for both asymmetric S100B monomers of all NMR conformers submitted.

c Sum of values for the peptide interactions with both asymmetric S100B monomers.

d Mean value for the peptide interactions with both asymmetric S100B monomers

## References

[1] Donato R (2001) S100: A multigenic family of calcium-modulated proteins of the EF-hand type with intracellular and extracellular functional roles. Int J Biochem Cell Biol 33: 637–668. 11390274 10.1016/s1357-2725(01)00046-2

[2] Donato R, Sorci G, Riuzzi F, Arcuri C, Bianchi R, (2009) S100B’s double life: Intracellular regulator and extracellular signal. Biochim Biophys Acta 1793: 1008–1022. 19110011 10.1016/j.bbamcr.2008.11.009

[3] Hachem S, Laurenson A, Hugnot JP, Legraverend C (2007) Expression of S100B during embryonic development of the mouse cerebellum. BMC Dev Biol 7: 17. 17362503 10.1186/1471-213X-7-17 PMC1832187

[4] Lin J, Blake M, Tang C, Zimmer D, Rustandi RR, (2001) Inhibition of p^53^ transcriptional activity by the S100B calcium-binding protein. J Biol Chem 276: 35037–35041. 11454863 10.1074/jbc.M104379200

[5] Lin J, Yang Q, Wilder PT, Carrier F, Weber DJ (2010) The calcium-binding protein S100B down-regulates p^53^ and apoptosis in malignant melanoma. J Biol Chem 285: 27487–27498. 20587415 10.1074/jbc.M110.155382 PMC2930747

[6] Tubaro C, Arcuri C, Giambanco I, Donato R (2010) S100B protein in myoblasts modulates myogenic differentiation via NF-kappaB-dependent inhibition of MyoD expression. J Cell Physiol 223: 270–282. 20069545 10.1002/jcp.22035

[7] Tubaro C, Arcuri C, Giambanco I, Donato R (2011) S100B in myoblasts regulates the transition from activation to quiescence and from quiescence to activation and reduces apoptosis. Biochem Biophys Acta 1813: 1092–1104. 21130124 10.1016/j.bbamcr.2010.11.015

[8] Liu J, Wang H, Zhang L, Xu Y, Deng W, (2011) S100B transgenic mice develop features of Parkinson’s disease. Arch Med Res 42: 1–7. 21376255 10.1016/j.arcmed.2011.01.005

[9] Schroeter ML, Abdul-Khaliq H, Sacher J, Steiner J, Blasig IE, (2010) Mood disorders are glial disorders: Evidence from in vivo studies. Cardiovasc Psychiatry Neurol 1: 780. 10.1155/2010/780645 PMC2878670 20585358

[10] Rothermundt M, Ahn JN, Jorgens S (2009) S100B in schizophrenia: An update. Gen Physiol Biophys 28 1: F76–81. 20093730

[11] Mocellin S, Zavagno G, Nitti D (2008) The prognostic value of serum S100B in patients with cutaneous melanoma: A meta-analysis. Int J Cancer 123: 2370–2376. 18752249 10.1002/ijc.23794

[12] Jiang W, Jia Q, Liu L, Zhao X, Tan A, (2011) S100B promotes the proliferation, migration and invasion of specific brain metastatic lung adenocarcinoma cell line. Cell Biochem Funct 29: 582–588. 21861268 10.1002/cbf.1791

[13] Jung K, Goerdt C, Lange P, Blocher J, Djukic M, (2011) The use of S100B and Tau protein concentrations in the cerebrospinal fluid for the differential diagnosis of bacterial meningitis: a retrospective analysis. Eur Neurol 66: 128–132. 21865761 10.1159/000330566

[14] Hearst SM, Walker LR, Shao Q, Lopez M, Raucher D, (2011) The design and delivery of a thermally responsive peptide to inhibit S100B- mediated neurodegeneration. Neurosci 197: 369–380. 10.1016/j.neuroscience.2011.09.025 PMC3210406 21958864

[15] Wilder PT, Lin J, Bair CL, Charpentier TH, Yang D, (2006) Recognition of the tumor suppressor protein p53 and other protein targets by the calcium-binding protein S100B. Biochim Biophys Acta 1763: 1284–1297. 17010455 10.1016/j.bbamcr.2006.08.024

[16] Rustandi RR, Drohat AC, Baldisseri DM, Wilder PT, Weber DJ (1998) The Ca2+-dependent interaction of S100B (beta beta) with a peptide derived from p53. Biochem 37: 1951–1960. 9485322 10.1021/bi972701n

[17] Fernandez-Fernandez MR, Veprintsev DB, Fersht AR (2005) Proteins of the S100 family regulate the oligomerization of p53 tumor suppressor. Proc Natl Acad Sci USA 102: 4735–4740. 15781852 10.1073/pnas.0501459102 PMC555715

[18] Ivanenkov VV, Jamieson GA, Gruenstein E, Dimlich RVW (1995) Characterization of S-100b binding epitopes. Identification of a novel target, the actin capping protein. CapZ J Biol Chem 270: 14651–14658. 7540176 10.1074/jbc.270.24.14651

[19] Bhattacharya S, Large E, Heizmann CW, Hemmings BA, Chazin WJ (2003) Structure of the Ca2+/S100B/NDR kinase peptide complex: insights into S100 target specificity and activation of the kinase. Biochemis 42: 14416–14426. 10.1021/bi035089a 14661952

[20] Wilder PT, Rustandi RR, Drohat AC, Weber DJ (1998) S100B (Beta-Beta) inhibits the protein kinase C-dependent phosphorylation of a peptide derived from p53 in a Ca^2+^-dependent manner. Protein Sci 7: 794–798. 9541413 10.1002/pro.5560070330 PMC2143941

[21] Duda T (2002) Ca (2+) sensor S100beta-modulated sites of membrane guanylate cyclase in the photoreceptor-bipolar synapse. EMBO J 21: 2547–2556. 12032068 10.1093/emboj/21.11.2547 PMC125384

[22] Duda T, Sharma RK (2004) S100B-modulated Ca^2+^-dependent ROS-GC1 transduction machinery in the gustatory epithelium: A new mechanism in gustatory transduction. FEBS Lett 577: 393–398. 15556616 10.1016/j.febslet.2004.09.089

[23] Baudier J, Mochly-Rosen D, Newton A, Lee SH, Koshland DE, (1987) Comparison of S100b protein with calmodulin: interactions with melittin and microtubule-associated tau proteins and inhibition of phosphorylation of tau proteins by protein kinase C. Biochem 26: 2886–2893. 3111527 10.1021/bi00384a033

[24] Cristovao JS (2018) The neuronal S100B protein is a calcium-tuned suppressor of amyloid-beta aggregation. Sci Adv 4: 1702. 10.1126/sciadv.aaq1702 PMC6025902 29963623

[25] Kazakov AS, Sokolov AS, Vologzhannikova AA, Permyakova ME, Khorn PA, (2017) Interleukin-11 binds specific EF-hand proteins via their conserved structural motifs. J Biomol Struct Dyn 35: 78–91. 26726132 10.1080/07391102.2015.1132392

[26] Stroth N, Svenningsson P (2015) S100B interacts with the serotonin 5- HT7 receptor to regulate a depressive-like behavior. Eur Neuropsychopharmacol 25: 2372–2380. 26499172 10.1016/j.euroneuro.2015.10.003

[27] Dempsey BR, Shaw GS (2011) Identification of calcium-independent and calcium-enhanced binding between S100B and the dopamine D2 receptor. Biochem 50: 9056–9065. 21932834 10.1021/bi201054x PMC3196243

[28] Hartman KG, Vitolo MI, Pierce AD, Fox JM, Shapiro P, (2014) Complex formation between S100B protein and the p90 ribosomal S6 kinase (RSK) in malignant melanoma is calcium-dependent and inhibits extracellular signal-regulated kinase (ERK)-mediated phosphorylation of RSK. J Biol Chem 289: 12886–12895. 24627490 10.1074/jbc.M114.561613 PMC4007476

[29] Gogl G, Alexa A, Kiss B, Katona G, Kovács M, (2016) Structural basis of ribosomal s6 kinase 1 (RSK1) inhibition by S100B protein: Modulation of the extracellular signal-regulated kinase (ERK) signaling cascade in a calcium-dependent way. J Biol Chem 291: 11–27. 26527685 10.1074/jbc.M115.684928 PMC4697148

[30] Ostendorp T, Leclerc E, Galichet A, Koch M, Demling N, (2007) Structural and functional insights into RAGE activation by multimeric S100B. EMBO J 26: 3868–3878. 17660747 10.1038/sj.emboj.7601805 PMC1952220

[31] Kligman D, Hilt DC (2013) The S100 protein family. Trends Biochem Sci 13: 437–43. 10.1016/0968-0004(88)90218-6 3075365

[32] Donato R (2010) Functions of S100 proteins. Curr Mol Med 13: 24–57. PMC3707951 22834835

[33] Smith S, Shaw GS (1998) A change-in-hand mechanism for S100 signalling. Biochem Cell Biol 76: 324–333. 9923701 10.1139/bcb-76-2-3-324

[34] Zimmer DB, Sadosky W, Weber DJ (2003) Molecular mechanisms of S100-target protein interactions. Microsc Res Tech 60: 552–559. 12645003 10.1002/jemt.10297

[35] Kilby PM, Eldik LJV, Roberts GC (1996) The solution structure of the bovine S100B protein dimer in the calcium-free state. Structure 4: 1041–1052. 8805590 10.1016/s0969-2126(96)00111-6

[36] Ostendorp T (2011) The crystal structures of human S100B in the zinc- and calcium-loaded state at three pH values reveal zinc ligand swapping. Biochim Biophys Acta 1813: 1083–1091. 20950652 10.1016/j.bbamcr.2010.10.006

[37] Malik S (2008) Analysis of the structure of human apo-S100B at low temperature indicates a unimodal conformational distribution is adopted by calcium-free S100 proteins. Proteins 73: 28–42. 18384084 10.1002/prot.22037

[38] Drohat AC, Baldisseri DM, Rustandi RR, Weber DJ (1998) Solution structure of calcium-bound rat S100B(betabeta) as determined by nuclear magnetic resonance spectroscopy. Biochem 37: 2729–2740. 9485423 10.1021/bi972635p

[39] Wilder PT, Varney KM, Weiss MB, Gitti RK, Weber DJ (2005) Solution structure of zinc- and calcium-bound rat S100B as determined by nuclear magnetic resonance spectroscopy. Biochemi 44: 5690–5702. 10.1021/bi0475830 15823027

[40] Charpentier TH, Wilder PT, Liriano MA, Varney KM, Pozharski E, (2008)Divalent metal ion complexes of S100B in the absence and presence of pentamidine. J Mol Biol 382: 56–73. 18602402 10.1016/j.jmb.2008.06.047 PMC2636698

[41] Charpentier TH, Wilder PT, Liriano MA, Varney KM, Zhong S, (2009) Small molecules bound to unique sites in the target protein binding cleft of calcium-bound S100B as characterized by nuclear magnetic resonance and X-ray crystallography. Biochem 48: 6202–6212 19469484 10.1021/bi9005754 PMC2804263

[42] Rezvanpour A, Shaw GS (2009) Unique S100 target protein interactions. Gen Physiol Biophys 1: F39–46. 20093725

[43] Gentil BJ, Delphin C, Mbele GO, Deloulme JC, Ferro M, (2001) The giant protein AHNAK is a specific target for the calcium- and zinc-binding S100B protein: potential implications for Ca^2+^ homeostasis regulation by S100B. J Biol Chem 276: 23253–23261. 11312263 10.1074/jbc.M010655200

[44] Rustandi RR, Baldisseri DM, Weber DJ (2000) Structure of the negative regulatory domain of p53 bound to S100B (betabeta). Nat Struct Biol 7: 570–574. 10876243 10.1038/76797

[45] Jensen JL, Indurthi VSK, Neau DB, Vetter SW, Colbert CL (2015) Structural insights into the binding of the human receptor for advanced glycation end products (RAGE) by S100B, as revealed by an S100B- RAGE-derived peptide complex. Acta Crystallogr D Biol Crystallogr 71: 1176–1183. 25945582 10.1107/S1399004715004216 PMC4427201

[46] Inman KG, Yang R, Rustandi RR, Miller KE, Baldisseri DM, (2002) Solution NMR structure of S100B bound to the high-affinity target peptide TRTK-12. J Mol Biol 324: 1003–1014. 12470955 10.1016/s0022-2836(02)01152-x

[47] Charpentier TH, Thompson LE, Liriano MA, Varney KM, Wilder PT, (2010) The effects of CapZ peptide (TRTK-12) binding to S100B-Ca2+ as examined by NMR and X-ray crystallography. J Mol Biol 396: 1227–1243. 20053360 10.1016/j.jmb.2009.12.057 PMC2843395

[48] McClintock KA, Shaw GS (2003) A novel S100 target conformation is revealed by the solution structure of the Ca^2+^-S100B-TRTK-12 complex. J Biol Chem 278: 6251–6257. 12480931 10.1074/jbc.M210622200

[49] Baudier J, Delphin C, Grunwald D, Khochbin S, Lawrence JJ, (1992) Characterization of the tumor suppressor protein p53 as a protein kinase C substrate and a S100b-binding protein. Proc Natl Acad Sci USA 89: 11627–11631. 1454855 10.1073/pnas.89.23.11627 PMC50606

[50] Albert KA (1984) Inhibition by calmodulin of calcium/phospholipid- dependent protein phosphorylation. Proc Natl Acad Sci USA 81: 3622–3625. 6233611 10.1073/pnas.81.12.3622 PMC345270

[51] Markowitz J, Chen I, Gitti R, Baldisseri DM, Pan Y, (2004) Identification and characterization of small molecule inhibitors of the calcium-dependent S100B-p53 tumor suppressor interaction. J Med Chem 47: 5085–5093. 15456252 10.1021/jm0497038

[52] Weber D (2010) In vitro screening and structural characterization of inhibitors of the S100B-p53 interaction. Int J High Throughput Screen 2010: 109–126. 21132089 10.2147/IJHTS.S8210 PMC2995924

[53] Capoccia E, Cirillo C, Marchetto A, Tiberi S, Sawikr Y, (2015) S100B-p53 disengagement by pentamidine promotes apoptosis and inhibits cellular migration via aquaporin-4 and metalloproteinase-2 inhibition in C6 glioma cells. Oncol Lett 9: 2864–2870. 26137161 10.3892/ol.2015.3091 PMC4473713

[54] Agamennone M, Cesari L, Lalli D, Turlizzi E, Conte RD, (2010) Fragmenting the S100B-p53 interaction: combined virtual/biophysical screening approaches to identify ligands. Chem Med Chem 5: 428–435. 20077460 10.1002/cmdc.200900393

[55] Cavalier MC (2016) Small molecule inhibitors of Ca^2+^-S100B reveal two protein conformations. J Med Chem 59: 592–608. 26727270 10.1021/acs.jmedchem.5b01369 PMC4732916

[56] Cavalier MC, Melville Z, Aligholizadeh E, Raman EP, Yu W, (2016) Novel protein-inhibitor interactions in site 3 of Ca^2+^ bound S100B as discovered by X-ray crystallography. Acta Crystallogr D Struct Biol 72: 753–760. 27303795 10.1107/S2059798316005532 PMC4908867

[57] Cavalier MC, Pierce AD, Wilder PT, Alasady MJ, Hartman KG, (2014) Covalent small molecule inhibitors of Ca^2+^ bound S100B. Biochem 53: 6628–6640. 25268459 10.1021/bi5005552 PMC4211652

[58] Krissinel E, Henrick K (2007) Inference of macromolecular assemblies from crystalline state. J Mol Biol 372: 774–797. 17681537 10.1016/j.jmb.2007.05.022

[59] Schaeffer L, Roy R, Humbert S, Moncollin V, Vermeulen W, (1993) LDNA repair helicase: A component of BTF2 (TFIIH) basic transcription factor. Sci 260: 58–63. 10.1126/science.8465201 8465201

[60] Fuss JO, Tainer JA (2011) XPB and XPD helicases in TFIIH orchestrate DNA duplex opening and damage verification to coordinate repair with transcription and cell cycle via CAK kinase. DNA Repair 10: 697–713. 21571596 10.1016/j.dnarep.2011.04.028 PMC3234290

[61] Bradsher J, Coin F, Egly JM (2000) Distinct roles for the helicases of TFIIH in transcript initiation and promoter escape. J Biol Chem 275: 2532–2538. 10644710 10.1074/jbc.275.4.2532

[62] Kim TK (2000) Mechanism of ATP-dependent promoter melting by transcription factor IIH. Sci 288: 1418–1422. 10.1126/science.288.5470.1418 10827951

[63] Coin F, Oksenych V, Egly JM (2007) Distinct roles for the XPB/p52 and XPD/p44 subcomplexes of TFIIH in damaged DNA opening during nucleotide excision repair. Mol Cell 26: 245–256. 17466626 10.1016/j.molcel.2007.03.009

[64] Fan L, Arvai AS, Cooper PK, Iwai S, Hanaoka F, (2006) Conserved XPB core structure and motifs for DNA unwinding: implications for pathway selection of transcription or excision repair. Mol Cell 22: 27–37. 16600867 10.1016/j.molcel.2006.02.017

[65] Kahanda D, DuPrez KT, Hilario E, McWilliams MA, Wohlgamuth CH, (2018) Application of electrochemical devices to characterize the dynamic actions of helicases on DNA. Anal Chem 90: 2178–2185. 29285929 10.1021/acs.analchem.7b04515 PMC5957534

[66] Plaschka C, Hantsche M, Dienemann C, Burzinski C, Plitzko J, (2006) Transcription initiation complex structures elucidate DNA opening. Nature 533: 353–358. 10.1038/nature17990 27193681

[67] Schilbach S, Hantsche M, Tegunov D, Dienemann C, Wigge C, (2007) Structures of transcription pre-initiation complex with TFIIH and mediator. Nature 551: 204–209. 10.1038/nature24282 PMC6078178 29088706

[68] Luo J, Cimermancic P, Viswanath S, Ebmeier CC, Kim B, (2015) Architecture of the human and yeast general transcription and DNA repair factor TFIIH. Mol Cell 59: 794–806. 26340423 10.1016/j.molcel.2015.07.016 PMC4560838

[69] Hilario E, Li Y, Nobumori Y, Liu X, Fan L (2013) Structure of the C- terminal half of human XPB helicase and the impact of the disease- causing mutation XP11BE. Acta Crystallogr D Biol Crystallogr 69: 237–246. 23385459 10.1107/S0907444912045040

[70] Coin F, Auriol J, Tapias A, Clivio P, Vermeulen W, (2004) Phosphorylation of XPB helicase regulates TFIIH nucleotide excision repair activity. EMBO J 23: 4835–4846. 15549133 10.1038/sj.emboj.7600480 PMC535092

